# An Exploratory Study of Medical Journal’s Twitter Use: Metadata, Networks, and Content Analyses

**DOI:** 10.2196/43521

**Published:** 2023-01-19

**Authors:** Donghun Kim, Woojin Jung, Ting Jiang, Yongjun Zhu

**Affiliations:** 1 Department of Library and Information Science, Yonsei University Seoul Republic of Korea; 2 Department of Library and Information Science, Sungkyunkwan University Seoul Republic of Korea

**Keywords:** medical journals, social networks, Twitter

## Abstract

**Background:**

An increasing number of medical journals are using social media to promote themselves and communicate with their readers. However, little is known about how medical journals use Twitter and what their social media management strategies are.

**Objective:**

This study aimed to understand how medical journals use Twitter from a global standpoint. We conducted a broad, in-depth analysis of all the available Twitter accounts of medical journals indexed by major indexing services, with a particular focus on their social networks and content.

**Methods:**

The Twitter profiles and metadata of medical journals were analyzed along with the social networks on their Twitter accounts.

**Results:**

The results showed that overall, publishers used different strategies regarding Twitter adoption, Twitter use patterns, and their subsequent decisions. The following specific findings were noted: journals with Twitter accounts had a significantly higher number of publications and a greater impact than their counterparts; subscription journals had a slightly higher Twitter adoption rate (2%) than open access journals; journals with higher impact had more followers; and prestigious journals rarely followed other lesser-known journals on social media. In addition, an in-depth analysis of 2000 randomly selected tweets from 4 prestigious journals revealed that *The Lancet* had dedicated considerable effort to communicating with people about health information and fulfilling its social responsibility by organizing committees and activities to engage with a broad range of health-related issues; *The New England Journal of Medicine* and *the Journal of the American Medical Association* focused on promoting research articles and attempting to maximize the visibility of their research articles; and *the British Medical Journal* provided copious amounts of health information and discussed various health-related social problems to increase social awareness of the field of medicine.

**Conclusions:**

Our study used various perspectives to investigate how medical journals use Twitter and explored the Twitter management strategies of 4 of the most prestigious journals. Our study provides a detailed understanding of medical journals’ use of Twitter from various perspectives and can help publishers, journals, and researchers to better use Twitter for their respective purposes.

## Introduction

### Background

Social media has become increasingly important in our everyday lives, as it is the means through which we acquire and share information. Individuals, corporations, organizations, and governments are increasingly using social media to broadcast information to various audiences. Social media use is also becoming increasingly prevalent in academia. Despite disciplinary differences, researchers use social media to discuss research ideas, share information and knowledge, and provide feedback on published materials [1]. Existing studies have aimed to measure the impact of scholarly articles [2] and educational resources [3] based on their mentions on social media. Social media–based indicators are known to reflect *impact* in a manner different from the traditional citation-based indicators such as the impact factor [2], though they complement each other [4].

Social media is also increasingly influencing medicine and health care [5]. For example, Twitter has been actively adopted by clinicians, specialized medical conferences, and academic medical departments for various purposes such as interacting with patients and health care professionals, sharing expertise and knowledge, and marketing services [6-8]. Medical conferences use Twitter hashtags to broadcast live virtual meetings, engage with meeting attendees, and promote collaboration [9]. Academic medical departments also use Twitter to share educational content, departmental awards, and accomplishments and to disseminate departmental research [10].

Medical journals have traditionally used news media to increase visibility [11-13]. As medical journals are mainly read by practicing doctors who have little time to read original research published in journals [14], increasing the visibility of medical journals through new communication channels is important to emphasize their value. With the rise of social media, an increasing number of medical journals are using social media to promote themselves and communicate with their readers [15]. Previous research has shown that medical journals with Twitter profiles have higher impact factors than those without Twitter profiles [16], and the number of Twitter followers of a medical journal is correlated with its impact factor [17]. Ranging from simple tasks such as sharing new articles and creating infographics to more complicated tasks such as hosting Twitter chats and web-based journal clubs, medical journals have used various strategies to promote themselves, engage with journal readers, and improve knowledge translation [18]. Several studies have also reported on the positive impact of social media on medical journals. For example, sharing of articles on Twitter increases page views and web traffic of medical journals [19-21]. Podcast- and infographic-based promotion of research articles on Twitter increases their Altmetric scores and abstract views [22]. In addition, organizing Twitter chats on the topic of general interest increases journals’ audiences and reach [23].

Although multiple studies have investigated how medical journals have used Twitter, our overall understanding remains limited, mainly because existing studies have focused on particular medical specialties, such as radiology [16] or urology [24], and have analyzed Twitter profiles and metadata such as the number of posts, followers, and followings of each journal. Therefore, little is known about the general adoption of Twitter by medical journals or their diverse social media management strategies, and more research is needed to understand medical journals’ use of Twitter and their social media management strategies from a global perspective by performing a broader scale and more in-depth analysis.

### Objectives

To fill this gap, we aimed to analyze all the available Twitter accounts of medical journals indexed by major indexing services by focusing on their social networks and content posted on social networks. Specifically, we created 3 research questions (RQs) that focused on Twitter profiles and metadata, the social networks of Twitter accounts, and Twitter content:

- RQ1: What are the adoption and use rates and the patterns of Twitter use of medical journals?
- RQ2: What are the structures of the social networks of medical journals’ Twitter accounts, and how do they interact on Twitter?
- RQ3: What are the major Twitter management strategies of medical journals, and how do these strategies differ among prestigious medical journals?

The remainder of this paper is organized as follows. In the Methods section, we introduce the data collection and preprocessing approaches as well as the data analysis. The following sections then interpret the results of the data analysis, discuss the major findings and limitations of the study, and finally conclude the paper.

## Methods

### Data

We collated all journal titles in the “Medicine” category of the SCIMago Journal Rank (SJR) [25], which was selected from the Journal Citation Reports of Clarivate Analytics because of its broader coverage of medical journals. As of March 2021, we collated 7322 medical journal titles, which were themselves based on Scopus data as of April 2020. By manually accessing each journal’s official website and performing a Google search, we collected their official Twitter accounts. Using the journals’ Twitter accounts, we crawled their metadata (ie, *accounts_name*, *joined_date*, *number of followers*, and *number of followings*) and tweets (ie, *tweet_contents*, *tweet_timestamp*, and *tweet_url*) using Python libraries Selenium [26] and Snscrape [27]. Table 1 presents these data in detail.

Table 1 shows that 46.8% (3427/7322) of journals did not have Twitter accounts, whereas 33.2% (2431/7322) of journals shared their Twitter accounts with other journals from the same publisher. As we were looking to analyze individual journals’ Twitter use in this study, we excluded journals with publisher accounts. Therefore, we were left with 19.8% (1450/7322) of journals that had independent Twitter accounts.

**Table 1:** Data description of all the journals in the “Medicine” category of SCIMago Journal Rank (N=7322).

Item	Values, n (%)
Journals with Twitter accounts	3895 (53.2)
Journals with unique Twitter accounts	1450 (19.8)
Journals with publisher Twitter accounts	2431 (33.2)
Journals with editor Twitter accounts	14 (0.19)
Journals without Twitter accounts	3427 (46.8)
Publishing countries among the journals	96 (1.31)
Publishers among the journals	2199 (30.03)

### Detailed Methods

#### Exploratory Analysis

To understand the various factors that could affect the adoption and use rates and patterns of Twitter use, we explored factors such as the SJR indicator, h-index, total documents (3 years), total citations (3 years), citations per document (2 years), country, established year, subject area, publisher, and subscription type. These factors were investigated to identify the differences in Twitter adoption and use rates among medical journals. Table 2 provides a description of the analyzed factors.

**Table 2:** Description of the analyzed factors.

Factor	Description
SCIMago Journal Rank indicator	Average number of weighted citations received in 2020 by articles published in the journal in the preceding 3 years
h-index	The number of articles cited at least “h” times
Total documents (3 years)	The number of journal documents published in the preceding 3 years
Total citations (3 years)	The number of journal citations in the preceding 3 years
Citations per document (2 years)	Average number of citations per document in the preceding 2 years
Country	Country of journal publication
Established year	Year of a journal’s establishment
Subject area	Journal’s specific subject categories according to Scopus classification
Publisher	Journal publisher
Subscription type	Open access or standard subscription-based publication

#### Network Analysis

Medical journals interact with each other on Twitter by following and being followed by other medical journals. By collecting all the followers of each medical journal, we constructed a social network for medical journals and collated their social interactions. Specifically, we explored the social networks of journals, publishers, and countries.

#### Content Analysis

The content of various tweets was investigated to understand the various social media management strategies of the medical journals. We began by manually reviewing a random sample of 1000 tweets out of the total tweets posted by 1450 journals and identified major categories related to their social media management strategies. Next, we reviewed 500 tweets from the accounts of each of the 4 most prestigious medical journals (*The Lancet, the New England Journal of Medicine [NEJM], the Journal of the American Medical Association [JAMA]*, and *the British Medical Journal [BMJ]*) to understand the differences in social media management strategies. A total of 2 graduate students and 1 information science expert manually reviewed the tweets using an annotation process (ie, reviewing, coding, and refining the schema), which continued until an appropriate coding schema was developed.

The interrater reliability reached a Cohen κ value of 0.763, which was considered a substantial agreement [28] after the annotators had classified 1000 tweets. If some tweets were annotated differently by the annotators, expert intervention was used to reach a consensus.

### Ethical Considerations

No ethics review was sought because the study only explored the medical journals’ use of publicly available data on social media and did not conduct any experiments on human subjects.

## Results

### Twitter Adoption and Use Rates

**Figure 1:**
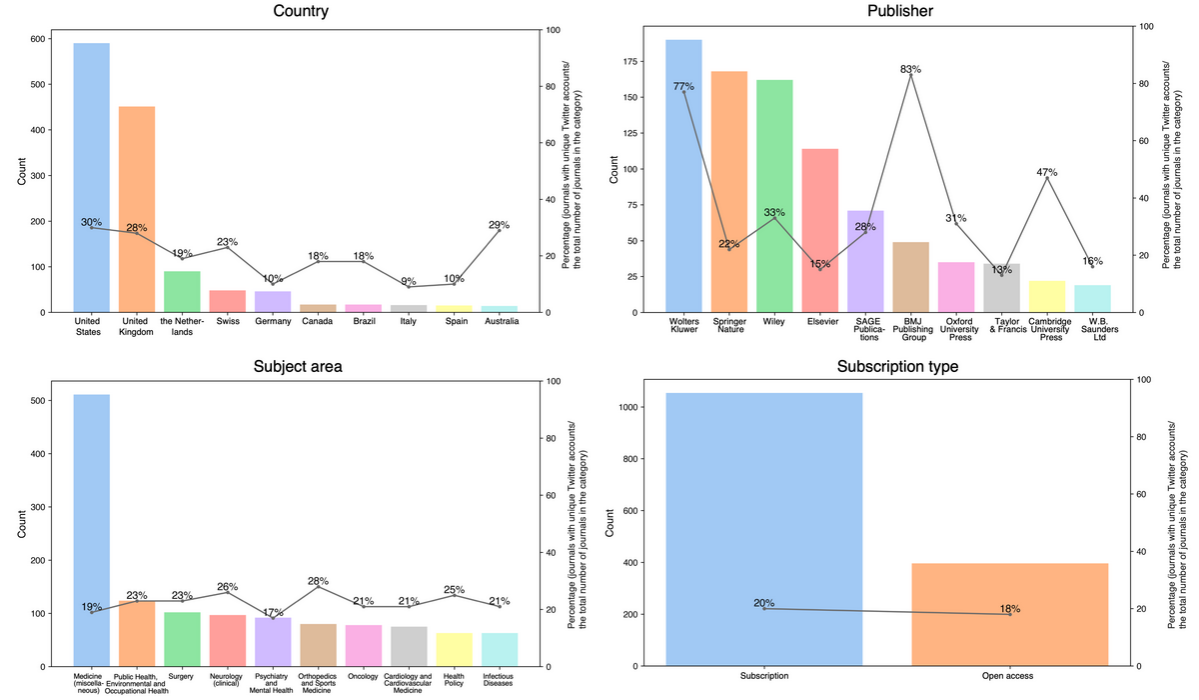
Distribution of countries, publishers, subject areas, and subscription types among journals with unique Twitter accounts. BMJ: British Medical Journal.

We compared the SJR indicator, h-index, total documents (ie, number of documents published in 2018, 2019, and 2020), total citations (ie, citations in 2021 received by documents published in 2018, 2019, and 2020), citations per document (ie, average citations per document in a 2-year period), established year, country, subject area, publisher, and the percentage of open access journals for journals with and without Twitter accounts in the group. A Student 2-tailed *t* test was performed against the 6 numerical variables (SJR indicator; h-index; total documents, 3 years; total citations, 3 years; citations per document, 2 years; and established year), with the differences between the 2 groups shown to be significant for all variables (all *P*<.001). Overall, journals with Twitter accounts had more publications and higher impact levels and were not as “old” as their non-Twitter counterparts. Figure 1 shows the distribution of countries, publishers, subject areas, and subscription types among journals with unique Twitter accounts.

The bars in Figure 1 represent the raw number of journals in a particular category, whereas the values above the dots represent the percentage of journals with unique Twitter accounts from the total number of journals in each category. For example, the upper-left figure in Figure 1 shows that 590 journals with unique Twitter accounts were published in the United States, which was 30.12% (590/1959) of all medical journals published in the country. Figure 1 shows that the United States and the United Kingdom were the 2 countries with the largest number and percentage of journals with unique Twitter accounts, followed by European countries such as the Netherlands, Swiss, and Germany. In addition, most journals published by Wolters Kluwer (190/246, 77.2%) and BMJ Publishing Group (49/59, 83%) had unique Twitter accounts, showing their highly active involvement in social media. Also, 46% (22/48) of Cambridge University Press’s journals, 34% (162/477) of Wiley’s journals, and 31.3% (35/112) of Oxford University Press’s journals have unique Twitter accounts. In most subject areas, 20.75% (2213/10,664) of the journals contained unique Twitter accounts. However, psychiatry and mental health had the lowest percentage of journals with unique Twitter accounts (92/538, 17.1%), whereas orthopedics and sports medicine had the highest percentage (80/281, 28.5%). Finally, the figure shows that subscription and open access journals had similar percentages of journals with unique Twitter accounts.

Among the journals with unique Twitter accounts, we investigated factors that could be related to Twitter use. Table 3 presents the correlation matrix (ie, the Pearson correlation coefficient) between 6 numerical journal variables (ie, SJR indicator; h-index; total documents, 3 years; total citations, 3 years; citations per document, 2 years; and established year) and 4 variables (ie, period of use [month], number of tweets, number of followings, and number of followers) related to Twitter use. As various journals joined Twitter at different times, the variables of Twitter use were appropriately normalized based on the period of Twitter use. For example, the table shows that the number of tweets was measured by the average number of tweets posted in a month, whereas the number of followers was calculated by dividing the total number of followers by the number of months since the date of account creation.

Table 3 shows 2 moderately positive correlations—one between the h-index and the number of followers and the other between the total citations (3 years) and the number of followers. We can see that journals with higher h-index values and those receiving more citations have more followers—a result that is consistent with the findings of a previous study where the author reported that the number of Twitter followers of medical journals correlated with their impact factors [17].

**Figure 2:**
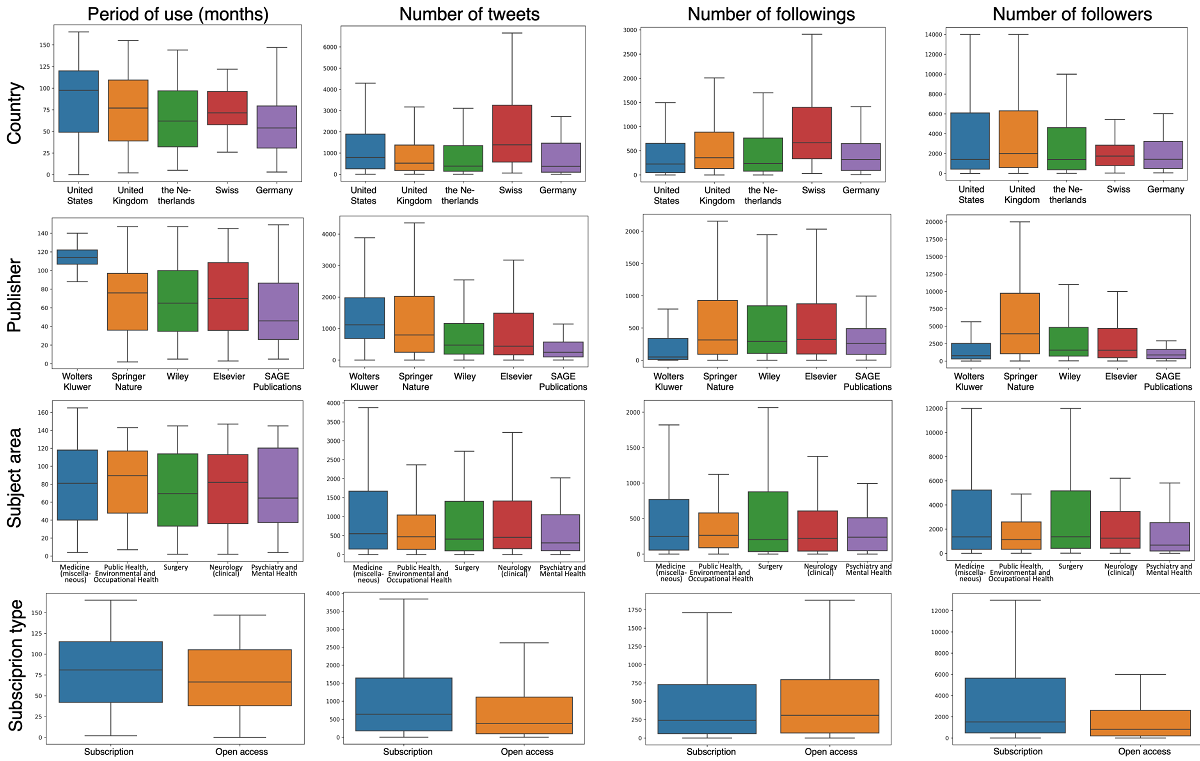
Distribution of countries, publishers, subject areas, and subscription types in relation to Twitter use variables.

Figure 2 shows the distribution of countries, publishers, subject areas, and subscription types related to the Twitter use variables.

**Table 3:** Correlation matrix between journal and Twitter use variables.

Journals			Period of use (month)		Tweets		Followings		Followers
**SCIMago Journal Rank indicator**									
	*r*	0.14		0.14		-0.02		0.31	
	*P* value	<.001		<.001		0.378		<.001	
**h-index**									
	*r*	0.20		0.25		-0.05		0.54	
	*P* value	<.001		<.001		0.080		<.001	
**Total documents (3 years)**									
	*r*	0.09		0.23		0.00		0.22	
	*P* value	<.001		<.001		0.894		<.001	
**Total citations (3 years)**									
	*r*	0.11		0.24		-0.01		0.50	
	*P* value	<.001		<.001		0.739		<.001	
**Citations per document (2 years)**									
	*r*	0.11		0.12		-0.01		0.26	
	*P* value	<.001		<.001		0.673		<.001	
**Established year**									
	*r*	−0.11		−0.10		0.06		−0.20	
	*P* value	<.001		<.001		0.021		<.001	

In each subfigure shown in Figure 2, we plotted the top 5 categories. Figure 2 shows that (1) Wolters Kluwer had used Twitter for longer than other publishers; (2) Swiss journals posted more tweets than the other countries featured in our study; (3) Swiss journals followed more journals, and Wolters Kluwer journals followed fewer journals than others; and (4) Springer Nature journals had more followers than journals from other publishers.

### Structure of the Social Network of Medical Journals’ Twitter Accounts

**Figure 3:**
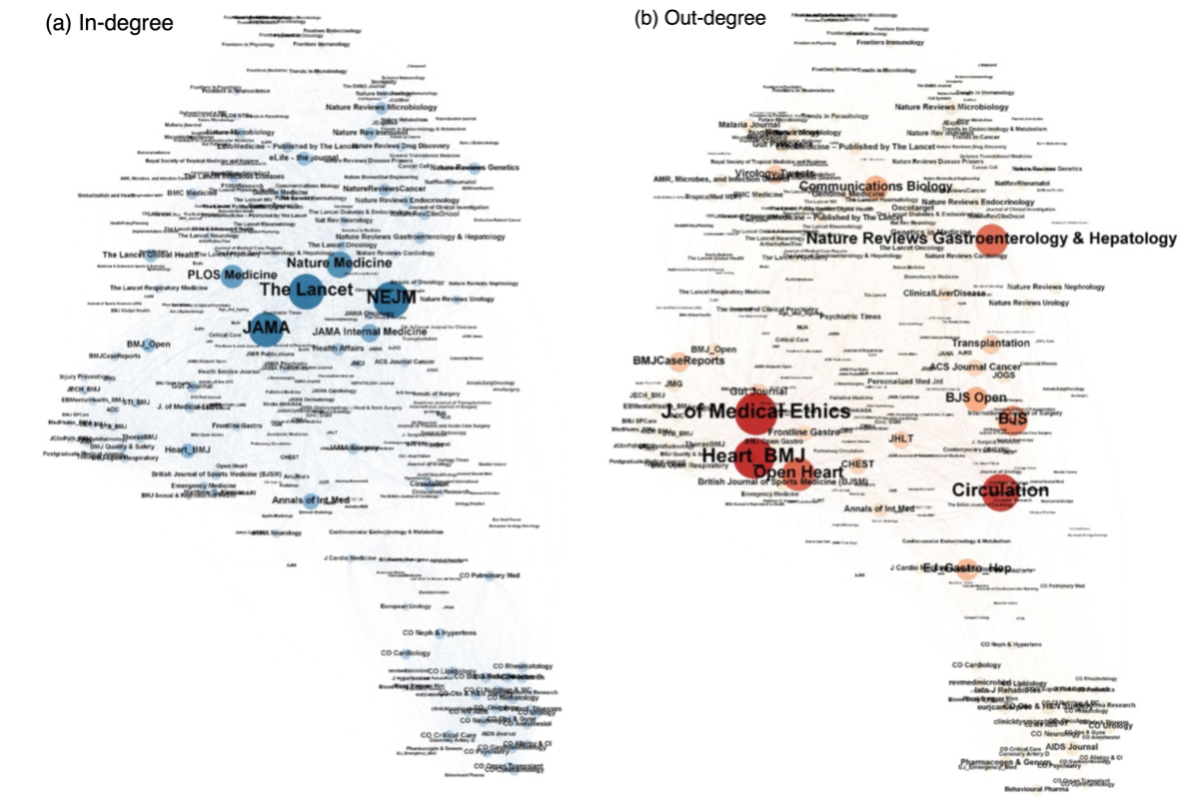
Social networks of medical journals’ Twitter accounts, (a) in-degree vs (b) out-degree. BJS: British Journal of Surgery, BMJ: British Medical Journal, JAMA: Journal of the American Medical Association, NEJM: New England Journal of Medicine.

We conducted a network analysis using Gephi 0.9.2 [29] to investigate the structures of the social networks created by the Twitter accounts of the medical journals. Among the 1450 journals, we only included journals that had accessible accounts and had >10 followers, which amounted to 1305 journals. Figure 3 shows 2 networks where the sizes and colors of the nodes and sizes of the labels are proportional to the number of followers (left: in-degree, which means the number of edges going into a node) and followings (right: out-degree, which means the number of edges going out of a node).

Figure 3 shows that the top 3 journals with the largest number of followers were all prestigious medical journals (*NEJM, The Lancet, and JAMA*). At the same time, the *Journal of Medical Ethics, Heart,* and *Circulation* were all major followers that do not have many followers themselves. We can also see that the more prestigious journals tend not to follow many other journals. A cluster of “current opinion” journals that follow each other was also found. Table 4 shows 2 lists—the top 10 journals with the highest in-degree and out-degree—with size, 2020 Journal Citation Reports impact factor, publisher, country, open access or subscription, and category.

Among the top 10 in-degree lists, journals with higher impact factors generally had more followers, as can be seen by the presence of *NEJM, The Lancet, JAMA, Nature Medicine, PLOS Medicine, JAMA Internal Medicine,* and *Annals of Internal Medicine.* Most journals in the top 10 list were about medicine (miscellaneous; 5/10, 50%), followed by internal medicine (3/10, 30%) and subscription journals (8/10, 80%); the journals were published by a variety of publishers mainly in the United States (6/10, 60%) and the United Kingdom (4/10, 40%). General and more prestigious medical journals had more followers than specialist journals. Among the top 10 listed in the out-degree, the most common category was cardiology and cardiovascular medicine, followed by surgery, gastroenterology, and hepatology. Although most of the journals in the list had impact factors <10, *Circulation* and *Nature Reviews Gastroenterology & Hepatology* had high impact factors of 30.0 and 46.8, respectively. BMJ Publishing Group, Springer Nature, Wiley, and Wolters Kluwer were the major publishers of the journals in the list.

**Figure 4:**
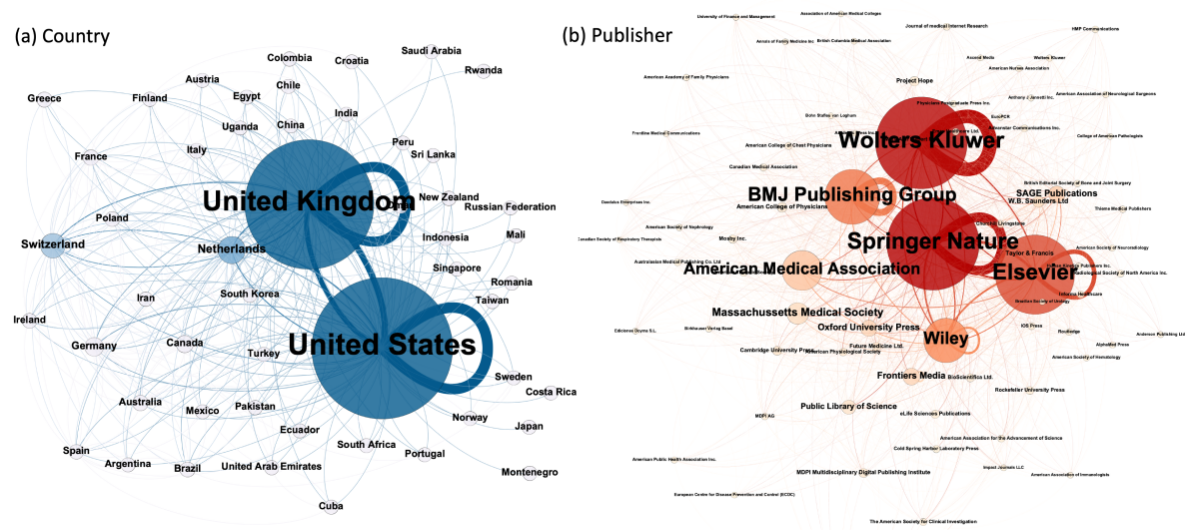
Social networks of (a) countries and (b) publishers of medical journals. BMJ: British Medical Journal.

Figure 4 shows the social networks among the countries and publishers of the journals analyzed in this study that have unique Twitter accounts. The size and color of the nodes shown in the figure are proportional to the size of the in-degree.

**Table 4:** Journals with the highest in-degree and out-degree proportions.

Journal			Size		Impact factor (2020)		Publisher		Country		Open access or Subscription		Category
**In-degree**													
	*The New England Journal of Medicine*	515		91.2		Massachusetts Medical Society		United States		Subscription		Medicine (miscellaneous)	
	*The Lancet*	466		79.3		Elsevier		United Kingdom		Subscription		Medicine (miscellaneous)	
	*The Journal of the American Medical Association*	435		56.3		American Medical Association		United States		Subscription		Medicine (miscellaneous)	
	*Nature Medicine*	242		53.4		Springer Nature		United Kingdom		Subscription		Medicine (miscellaneous)	
	*PLOS Medicine*	192		11.1		Public Library of Science		United States		Open access		Medicine (miscellaneous)	
	*JAMA Internal Medicine*	164		21.9		American Medical Association		United States		Subscription		Internal medicine	
	*Annals of Internal Medicine*	135		25.4		American College of Physicians		United States		Subscription		Internal medicine and medicine (miscellaneous)	
	*Health Affairs*	132		6.3		Project Hope		United States		Subscription		Health policy and medicine (miscellaneous)	
	*Heart*	104		6.0		BMJ^a^ Publishing Group		United Kingdom		Subscription		Cardiology and cardiovascular medicine	
	*eLife*	93		8.1		eLife Sciences Publications		United Kingdom		Open access		Medicine (miscellaneous)	
**Out-degree**													
	*Journal of Medical Ethics*	96		2.9		BMJ Publishing Group		United Kingdom		Subscription		Health policy	
	*Heart*	93		6.0		BMJ Publishing Group		United Kingdom		Subscription		Cardiology and cardiovascular medicine	
	*Circulation*	88		30.0		Wolters Kluwer		United States		Subscription		Cardiology and cardiovascular medicine and physiology (medical)	
	*Open Heart*	81		N/A^b^		BMJ Publishing Group		United Kingdom		Open access		Cardiology and cardiovascular medicine	
	*British Journal of Surgery*	80		3.4		Wiley		United States		Subscription		Surgery	
	*Nature Reviews Gastroenterology & Hepatology*	79		46.8		Springer Nature		United Kingdom		Subscription		Gastroenterology and hepatology	
	*British Journal of Surgery Open*	66		3.4		Wiley		United Kingdom		Open access		Surgery	
	*European Journal of Gastroenterology & Hepatology*	62		2.6		Wolters Kluwer		United States		Subscription		Gastroenterology and hepatology	
	*Communications Biology*	61		6.3		Springer Nature		United States		Open access		Medicine (miscellaneous)	
	*Chest*	60		9.4		American College of Chest Physicians		United States		Subscription		Cardiology and cardiovascular medicine, critical care and intensive care medicine, and pulmonary and respiratory medicine	

^a^BMJ: British Medical Journal.

^b^N/A: not applicable.

Unsurprisingly, the United States and the United Kingdom were the 2 countries that published the largest number of journals, followed by the Netherlands and Swiss. Springer Nature, Wolters Kluwer, Elsevier, BMJ Publishing Group, Wiley, and American Medical Associations were the 6 biggest publishers in terms of the number of followers. SAGE Publications—the fifth-largest publisher in terms of the number of published medical journals—had a smaller number of followers. However, the BMJ Publishing Group, which publishes less than half the number of journals published by Wiley (Figure 1), had more followers. Another notable example is the American Medical Association, which publishes <20 journals but has a comparable number of followers to that of Wiley, which publishes >150 journals. From the loops in the nodes, we can see that journals tend to follow journals from the same publisher rather than journals from other publishers.

### Exploration of Social Media Management Strategies

To understand the various purposes of Twitter use, we crawled 2,769,939 historical tweets posted by the medical journals analyzed in this study and reviewed a random sample of 1000 tweets.

In total, 15 categories were identified, and their proportions, in descending order, are listed in Table 5.

As shown in Table 5, most tweets from medical journals focused on introducing new articles or new issues. Most of these tweets simply contained a description of articles or journals, although some contained podcasts or blog posts to aid the understanding of the original articles. Medical journals also tended to use Twitter to provide health information to help increase public health literacy. They also used Twitter as a discussion channel to discuss certain health issues such as COVID-19 or childhood obesity with their readers. Occasionally, they hosted live Twitter chat events, tweeted questions (eg, “What resources can educate people on childhood obesity?”), and interacted with others in the comments.

On the basis of the identified categories, we compared the Twitter management strategies of 4 prestigious journals (*The Lancet, NEJM, JAMA, and BMJ*) to determine whether there were any differences. We reviewed 500 tweets from each of the 4 journals, and the results are presented in Table 6.

Among the identified 15 categories in Table 5, a total of 14 (except for “Introductions to research projects”) appeared in the examined tweets from the 4 prestigious medical journals. Table 6 shows that the 4 prestigious medical journals mainly tweeted descriptions of research articles or perspectives or reviews or case reports along with links to the full-text versions and descriptions of journals’ new issues or research collections. This finding is the same as that obtained when we analyzed the 1000 tweets shown in Table 5.

**Table 5:** Fifteen categories of Twitter management strategies (N=1000).

Category	Example	Values, n (%)
Descriptions of research articles or perspectives or reviews or case reports along with links to full-text versions	“Understanding the strengths and limitations of established and emerging techniques of pediatric lung MRI can allow practitioners to select and combine the optimal techniques. https://ajronline.org/doi/abs/10.2214/AJR.20.23104”	604 (60.4)
Descriptions of journals’ new issues or research collections	“Is This the Kind of Country We Want to Be? http://dlvr.it/CnV5Pp #BlogsCouchinCrisis”	89 (8.9)
News about nonmedical or health-related information	“Three teens were among many shot in Kansas City during a violent weekend. http://emsworld.com/11501544”	75 (7.5)
Notices or reports of academic conferences	“After @Peter_Ashman handles the intros @fgodlee delivers a ‘state of the nation’ on data sharing & patient partnerships #bmjeds14”	69 (6.9)
Health information	“@WHO announces list of eight diseases that may spark #publichealth emergency, as #Ebola outbreak may occur soon.”	59 (5.9)
Description of research articles with links to explanatory blog posts	“Simple exercise routines may outweigh the threats of air pollution, study shows. http://bit.ly/1WBw7F9”	30 (30)
Journal news	“With 9.499 citations, @ejsotweets new IF rises to 3.959 from 3.379 (2018)! We are strongly committed to clinical research and all aspects of surgical oncology which can advance the care of our patients”	24 (2.4)
Podcasts for specific health topics	“Video interview with @PaulLikeMe discussing patient-led research https://youtube.com/watch?v=XQZ5M9oLkXw&feature=youtu.be @patientslikeme #personalizedMed”	12 (1.2)
Discussions of health-related questions	“Do you think we can extrapolate these findings to men who do not suffer from gout & suggest they take allopurinol? What’s your take?”	12 (1.2)
Live Twitter chats for discussing health topics	“8 p.m. U.K. time tonight #BMJLeaderchat #Kindness & #Compassion in #leadership during #coronavirus”	10 (1)
Podcasts for research articles	“Listen on #Soundcloud to last week's trending Oncotarget paper: ‘Genomic markers of #midostaurin drug sensitivity in FLT3 mutated and FLT3 wild-type acute myeloid #leukemia patients’ #medEd #oncology #cancer #medicine”	8 (0.8)
Researcher introductions	“One of the stars of sport & exercise medicine in the world. Dr. Margot Putukian, MD. @MPutukian @MLS @Princeton”	4 (0.4)
Surveys of journal management strategies	“Take our ACS Open Access iPad Survey and win a cool fan! #ACSDallas”	2 (0.2)
Introductions to research projects	“The NFL is creating a partnership with researchers at Boston University who are studying the long-term effects of... http://bit.ly/6zGRaa”	1 (0.1)
Descriptions of research norms or ethics	“Plagiarism is the most heard word in scholarly publishing. Some papers are rejected only based on plagiarism. I am sharing herewith a link that will be useful to get insights about plagiarism: * Ethics of plagiarism *... http://ithenticate.com/resources/papers”	1 (0.1)

**Table 6:** The Twitter management strategies of 4 prestigious medical journals.

Category	The Lancet (n=500), n (%)	NEJM^a^ (n=500), n (%)	JAMA^b^ (n=500), n (%)	BMJ^c^ (n=500), n (%)
Descriptions of research articles or perspectives or reviews or case reports along with links to full-text versions	36 (72)	386 (77.2)	415 (83)	267 (53.4)
Descriptions of journals’ new issues or research collections	49 (9.8)	34 (6.8)	6 (1.2)	29 (5.8)
Descriptions of research articles with links to explanatory blog posts	0 (0)	16 (3.2)	3 (0.6)	11 (2.2)
Notices or reports of academic conferences	40 (8)	11 (2.2)	21 (4.2)	9 (1.8)
Infographics for research articles	1 (0.2)	1 (0.2)	1 (0.2)	4 (0.8)
Podcasts for research articles	5 (1)	32 (6.4)	49 (9.8)	2 (0.4)
Podcasts for specific health topics	13 (2.6)	12 (2.4)	1 (0.2)	4 (0.8)
Journal news	4 (0.8)	1 (0.2)	0 (0)	22 (4.4)
Health information	2 (0.4)	3 (0.6)	3 (0.6)	103 (20.6)
Researcher introductions	11 (2.2)	0 (0)	0 (0)	7 (1.4)
Discussions of health-related questions	0 (0)	0 (0)	1 (0.2)	0 (0)
Live Twitter chats for discussing health topics	6 (1.2)	0 (0)	0 (0)	3 (0.6)
Surveys of journal management strategies	0 (0)	0 (0)	0 (0)	1 (0.2)
News about nonmedical or health-related information	0 (0)	1 (0.2)	0 (0)	8 (1.6)
Descriptions of research norms or ethics	2 (0.4)	0 (0)	0 (0)	0 (0)
Other	7 (1.4)	3 (0.6)	0 (0)	30 (6)

^a^NEJM: New England Journal of Medicine.

^b^JAMA: Journal of the American Medical Association.

^c^BMJ: British Medical Journal.

In contrast, we found that each journal has its own unique Twitter management strategy. Compared with the other 3 journals, *The Lancet* had more tweets in the categories of *notices or reports of academic conferences* (40/500, 8% tweets), *podcasts for specific health topics* (13/500, 2.6% tweets), *researcher introductions* (11/500, 2.2% tweets), and *Twitter live chats* (6/500, 1.2% tweets) for discussing health topics. *The Lancet* appears to be more active in sharing information about academic conferences or its researchers and communicating with its readers to discuss and share health information. In addition to the 15 categories, *The Lancet* also tweeted about its public activities (6/500, 1.2% tweets) such as organizing committees and activities to deal with a broad range of health-related issues (eg, air or soil or water pollution and malaria). Currently, *The Lancet* is running 88 committees for global health and clinical issues [30].

*NEJM* had a higher percentage of tweets in categories such as *podcasts for research articles* (32/500, 6.4% tweets), *specific health topics* (12/500, 2.4% tweets), and *descriptions of research articles with links to explanatory blog posts* (16/500, 3.2% tweets) than the other 3 journals. *NEJM* attempts to help people understand research articles or disseminate specific health information faster and more easily than others. In addition to the 15 categories, *NEJM* tweeted about its web-based medical community—NEJM Resident 360 (3/500, 0.6% tweets)—to encourage residents to participate in various health-related discussions and knowledge exchanges that take place in the web-based community.

Compared with the other 3 journals, tweets introducing and promoting articles accounted for 83% (415/500) tweets of all tweets posted by *JAMA*. In addition, *JAMA* tweeted more frequently for *podcasts for research articles* (49/500, 9.8% tweets) than the others. These findings show that *JAMA* is primarily focused on promoting research articles.

Although it introduced research articles less frequently than the other 3 journals, *BMJ* had many more tweets providing *health information* (103/500, 20.6% tweets), which may be of interest to the general public. In addition, *BMJ* discussed various social problems (24/500, 4.8% tweets) such as racial discrimination in medical institutions, tobacco laws, and laws surrounding other drugs in low-income countries. These findings seem to indicate that *BMJ* uses Twitter not only to provide health information but also to increase social awareness of the field.

Furthermore, to understand the social media use of the 4 prestigious journals regarding the COVID-19 pandemic, we explored their tweets related to COVID-19 written in 2020. Overall, 32.05% (4237/13,222) of the tweets written in 2020 by the 4 journals were related to COVID-19. Among the 4 journals, *JAMA* (1219/3289, 37.06%) had the highest proportion of COVID-19–related tweets, followed by *The Lancet* (505/1398, 36.12%), *NEJM* (768/2408, 31.89%), and *BMJ* (1745/6127, 24.48%). The 4 journals devoted significant efforts to disseminating information about overcoming the pandemic. We reviewed 100 randomly sampled COVID-19–related tweets and found a broad range of topics including the introduction of COVID-19–related research articles, risk factors for COVID-19 infection, global data on confirmed COVID-19 cases, relationship between mental health and COVID-19, economic impacts of COVID-19, COVID-19 vaccines, and COVID-19 sequelae.

## Discussion

### Principal Findings

In this study, we investigated how medical journals use Twitter by analyzing the metadata, networks, and content of their Twitter accounts. Among the 7322 journals investigated, only 1450 (19.8%) had unique Twitter accounts. We also found that journals with Twitter accounts had a significantly greater number of publications and a higher impact than their counterparts that did not use Twitter. Journals with a higher impact may be more active in promotional aspects and would therefore be more likely to use social media such as Twitter. This active use of social media may improve the visibility of journals in the academic community and provide their articles with a higher probability of being read and subsequently cited. However, other factors could also be at play, and the study’s design was not sufficient to make any causal claims.

The journals of some publishers showed higher percentages of Twitter use. For example, 77% (190/246) and 83% (49/59) of journals published by Wolters Kluwer and the BMJ Publishing Group, respectively, had unique Twitter accounts, which shows their active involvement on social media. This is vastly greater than most other publishers who have Twitter adoption rates of ≤30%. Although Wolters Kluwer, Springer Nature, and Wiley all published similar numbers of medical journals, there were huge differences between their Twitter adoption rates (77%, 22%, and 34%, respectively). These 3 publishers created their Twitter accounts in January 2009, February 2011, and March 2010, respectively. Wolters Kluwer was undoubtedly an early adopter of Twitter and encouraged its journals to use the medium. Among the investigated subject areas, psychiatry and mental health had the lowest Twitter adoption rate (17%), whereas orthopedics and sports medicine had the highest Twitter adoption rate (28%). Although we assumed that open access journals would be more aggressive in their Twitter adoption rate, as one of their goals was to reduce access barriers to research and increase their audience, subscription journals actually showed a slightly higher (2%) Twitter adoption rate. This is because subscription journals have a more established business model and consistent revenue and may therefore have the financial resources to maintain several social media accounts.

Medical journals and their publishers were compared regarding Twitter use statistics. Journals with a higher impact in terms of the h-index and citations had more followers. Although citation-based impact measures are not sufficient for comparatively measuring journals’ values, they may act as an important indicator of when journals decide to follow each other, as journals tend to be more willing to follow high-impact journals. Prestigious medical journals such as *NEJM, The Lancet,* and *JAMA* had the largest number of followers on Twitter, although these journals rarely followed other less well-known journals. Generally, journals with large number of followers did not overlap with other journals that had a large number of followings, and the former had a higher citation-based impact than the latter.

We found that Wolters Kluwer had used Twitter for much longer than other publishers, SAGE Publications journals posted fewer tweets than others, and Springer Nature journals had more followers than others. However, in the publisher-level network, Springer Nature had a relatively small number of followers, which may indicate that it had a large number of followers from a relatively small number of publishers. The BMJ Publishing Group and American Medical Association were the 2 publishers with the highest publisher-level network followers, despite publishing only a relatively small number of journals, thereby showing the importance of these 2 publishers in this field. Therefore, these findings show that at the publisher-level social network, quality may be more important than the quantity of published journals.

We identified 15 major categories of Twitter management strategies used by medical journals. Among them, the promotion of articles and the promotion of journals’ new issues or collections were the main reasons for medical journals using Twitter. In addition to research-related purposes, they also tweeted to provide health information and live Twitter chats, which may help increase public health literacy.

Of the 4 prestigious medical journals compared in this study (*The Lancet, NEJM, JAMA,* and *BMJ*), we found that each had its own unique characteristics. *The Lancet* dedicated more effort to communicating with the general public about health information and aimed to fulfill its social responsibility as a major scientific journal by organizing committees and activities to discuss a broad range of health-related issues. *NEJM* and *JAMA* focused primarily on the promotion of research articles and tried to maximize their research articles’ visibility to reach more audiences by using various tools and methods. *BMJ*, in contrast, provided many health information articles to increase the public’s understanding and discussed various health-related social problems regarding increased social awareness of the field. All the 4 journals devoted significant efforts in disseminating information about overcoming the pandemic.

### Limitations

This study has a few limitations. First, we only collected the medical journals indexed in SJR, which is a major academic database. Therefore, other journals that were not indexed in SJR were excluded. As a result, the findings of this study should be considered only within this specific context. Second, regarding content analysis, we were unable to conduct an exhaustive investigation of the types of content posted by medical journals and their major purposes for using Twitter because of the large number of tweets. Instead, we randomly sampled 3000 tweets, and the reported results were based on the analysis of the sample. Finally, Twitter adoption and the use of data are dynamic; even at the time of writing, there may be many journals creating Twitter accounts, writing posts, and following others. Therefore, the results of this study provide a snapshot of a historical time and may change in the future.

### Conclusions

In this study, we analyzed the Twitter accounts of major medical journals indexed by SJR. Specifically, we investigated the Twitter use of 1450 medical journals by analyzing the metadata, social network, and content of their respective Twitter accounts. We found that journals with Twitter accounts had significantly more publications and a higher impact than their counterparts without a Twitter account. Journals with a higher impact in terms of h-index and citations had more followers than those with a lower impact. Prestigious medical journals such as *NEJM, The Lancet*, and *JAMA* have the largest number of followers, although journals with large numbers of followers did not overlap with other journals with equally large numbers of followings. The former has a higher citation-based impact than the latter. Through content analysis, we identified 15 major categories of medical journals’ Twitter management strategies. Finally, we compared the Twitter management strategies of the 4 prestigious medical journals and reported their unique characteristics. Our study provides a detailed understanding from various perspectives of how medical journals use Twitter, and it is hoped that this study can help publishers, journals, and researchers use Twitter better for various purposes.

## Abbreviations

BMJ: British Medical Journal
JAMA: Journal of the American Medical Association
NEJM: New England Journal of Medicine
RQ: research question
SJR: SCIMago Journal Rank
